# Mesenchymal Stromal Cells and Their Secretome: New Therapeutic Perspectives for Skeletal Muscle Regeneration

**DOI:** 10.3389/fbioe.2021.652970

**Published:** 2021-05-13

**Authors:** Martina Sandonà, Lorena Di Pietro, Federica Esposito, Alessia Ventura, Antonietta Rosa Silini, Ornella Parolini, Valentina Saccone

**Affiliations:** ^1^Istituto di Ricovero e Cura a Carattere Scientifico (IRCCS), Fondazione Santa Lucia, Rome, Italy; ^2^Dipartimento di Scienze della Vita e Sanità Pubblica, Università Cattolica del Sacro Cuore, Rome, Italy; ^3^Centro di Ricerca “E. Menni”, Fondazione Poliambulanza – Istituto Ospedaliero, Brescia, Italy; ^4^Fondazione Policlinico Universitario “Agostino Gemelli” IRCCS, Rome, Italy

**Keywords:** mesenchymal stromal cells, secretome, extracellular vesicles, muscle, muscle regeneration, atrophy, muscular dystrophy

## Abstract

Mesenchymal stromal cells (MSCs) are multipotent cells found in different tissues: bone marrow, peripheral blood, adipose tissues, skeletal muscle, perinatal tissues, and dental pulp. MSCs are able to self-renew and to differentiate into multiple lineages, and they have been extensively used for cell therapy mostly owing to their anti-fibrotic and immunoregulatory properties that have been suggested to be at the basis for their regenerative capability. MSCs exert their effects by releasing a variety of biologically active molecules such as growth factors, chemokines, and cytokines, either as soluble proteins or enclosed in extracellular vesicles (EVs). Analyses of MSC-derived secretome and in particular studies on EVs are attracting great attention from a medical point of view due to their ability to mimic all the therapeutic effects produced by the MSCs (i.e., endogenous tissue repair and regulation of the immune system). MSC-EVs could be advantageous compared with the parental cells because of their specific cargo containing mRNAs, miRNAs, and proteins that can be biologically transferred to recipient cells. MSC-EV storage, transfer, and production are easier; and their administration is also safer than MSC therapy. The skeletal muscle is a very adaptive tissue, but its regenerative potential is altered during acute and chronic conditions. Recent works demonstrate that both MSCs and their secretome are able to help myofiber regeneration enhancing myogenesis and, interestingly, can be manipulated as a novel strategy for therapeutic interventions in muscular diseases like muscular dystrophies or atrophy. In particular, MSC-EVs represent promising candidates for cell free-based muscle regeneration. In this review, we aim to give a complete picture of the therapeutic properties and advantages of MSCs and their products (MSC-derived EVs and secreted factors) relevant for skeletal muscle regeneration in main muscular diseases.

## Introduction

Mesenchymal“stromal” cells (MSCs) are adult, multipotent non-hematopoietic stem cells (Dominici et al., 2006). Over the last years, MSCs have emerged as a promising tool for cell therapy due to numerous features: their ability to self-renew and differentiate into several cell lineages, their ability to migrate to target tissues, and their immunomodulatory and anti-fibrotic properties, which can be attributed to their ability to secrete a plethora of biologically active molecules.

MSCs and/or mesenchymal-like cells can be isolated from numerous adult and perinatal tissues. MSCs were originally extracted from the bone marrow (Friedenstein et al., 1974) but have now been isolated from different sources: skeletal muscle (Kisiel et al., 2012), adipose tissue (Zuk et al., 2002), synovial membranes (De Bari et al., 2001), dental pulp (Rajendran et al., 2013), periodontal ligaments (Otabe et al., 2012), cervical tissue (Montesinos et al., 2013), peripheral blood (Longhini et al., 2019), suture mesenchyme of the skull (Lattanzi et al., 2013; Di Pietro et al., 2020), menstrual blood (Ren et al., 2016), perinatal tissues (Silini et al., 2015), and fetal blood (Campagnoli et al., 2001).

The International Society for Cellular Therapy (ISCT) established the minimal criteria necessary to identify MSCs: plastic adherence; positive for CD105, CD73, and CD90 surface markers; and negative (<2% expression) for CD11b, CD14, CD34, CD45, CD79a or CD19, and HLA class II (Dominici et al., 2006). Moreover, as multipotent cells, they must have the ability to differentiate into osteoblasts, adipocytes, and chondroblasts in *in vitro* differentiation conditions (Dominici et al., 2006). According to a few studies, MSCs can also express embryonic stem cell markers, such as Oct-4, Rex-1, and Sox-2 (Izadpanah et al., 2006; Riekstina et al., 2009). In 2008, similar criteria were established for MSCs from fetal membranes, with the addition that MSCs from fetal membranes must be of fetal origin (Parolini et al., 2008).

MSCs have gained much attention due to their *in vitro* and *in vivo* immunoregulatory capabilities that make them useful as guardians against excessive inflammatory responses (Prockop and Youn Oh, 2012; Magatti et al., 2017). Their protective and regenerative properties, as well as their immunoregulation skills, make them a valuable therapeutic strategy both for regenerative medicine and for the treatment of disorders characterized by alterations of the immune system.

MSCs have been shown to exert most of their effects through the release of molecules with paracrine and anti-inflammatory effects (Galderisi and Giordano, 2014). For this reason, more recently, different studies have focused on the secretome of MSCs, the set of biologically active molecules and extracellular vesicles (EVs) that these cells release. The secretome is nowadays considered a possible substitute for MSCs in cell therapy due to its comparable ability to enhance/favor tissue regeneration and modulate the immune response (Eleuteri and Fierabracci, 2019).

A hallmark in the study of the MSC secretome [or conditioned medium (CM)] was reached by Timmers and collaborators who fractionated the CM-MSC and discovered that the 50- to 200-nm component was the one with the most activity (Timmers et al., 2007). Subsequent characterization studies identified this fraction as EVs, which are lipid endogenous nanoparticles that mediate the transfer of their content across cellular boundaries (Guescini et al., 2010; Andaloussi et al., 2013; Romancino et al., 2013; Forterre et al., 2014).

EV is the generic term accepted by the International Society for Extracellular Vesicles (ISEV) to describe vesicles, characterized by the absence of a nucleus and the inability to replicate, that are released by cells into the extracellular space (Théry et al., 2018).

EVs are found in several biological fluids such as blood, urine, saliva, amniotic fluid, and milk (Iraci et al., 2016), and they can interact with the recipient cell by direct binding or ligand–receptor binding (Kahroba et al., 2019). EVs released by cells can be classified into different subtypes based on their physical features such as size or density. Small EVs (sEVs) have a typical size lower than 200 nm, while medium/large vesicles are characterized by a size greater than 200 nm. The classification of EVs is also based on their biochemical composition: the presence of transmembrane or glycosylphosphatidylinositol (GPI)-anchored proteins (e.g., CD63, CD81, and MHC class I), cytosolic proteins or periplasmic proteins (e.g., TSG101, Flotillin-1, Alix, and HSP-70), and proteins associated with non-EV structures (e.g., albumin and ApoA1/2).

EVs are characterized by a specific cargo composed of mRNAs, microRNAs (miRs), proteins, or DNA. This genetic material is protected by the oxidative extracellular environment and can be shuttled to distant cells in order to modulate the repair of damaged tissue (Cantaluppi et al., 2012; Borges et al., 2013).

In this review, we summarize the current research in the use of MSCs and of their secreted factors as alternative therapeutic strategies to improve skeletal muscle regeneration in the context of several muscle diseases. The MSC immune-modulatory and anti-fibrotic properties represent an attractive strategy to counteract the progression of chronic and in some cases lethal muscle diseases by reducing inflammation and fibrosis and stimulating the regenerative potential of muscle stem cells.

## Therapeutic Potential of Mesenchymal Stromal Cells in Skeletal Muscle Regeneration

The skeletal muscle is an adaptive tissue able to regenerate following damage due to trauma or genetic conditions. A complex stem cell niche resides in the muscle in which several cell types cooperate to regulate the balance between quiescence/activation/differentiation of the resident muscle stem cells, namely, satellite cells (MuSCs). In recent years, the role of other cell types has emerged, including inflammatory cells (Kharraz et al., 2013; Tidball et al., 2014) and mesenchymal cells residing in the muscle interstitium named fibro-adipogenic progenitors (FAPs), which maintain the homeostasis of the muscle niche and cooperate to support muscle regeneration (Mitchell et al., 2010; Uezumi et al., 2010, 2011; Malecova and Puri, 2012). For this reason, the muscle niche is a complex setting with different protagonists: FAPs play a pivotal role in coordinating tissue regeneration by supporting MuSC activity and by cooperating with inflammatory cells that mediate the activation of the regenerative response (Mozzetta et al., 2013; Madaro et al., 2018). During acute muscle injury, the myogenic program is properly activated, and inflammatory and muscle resident cells play a central role in modulating repair and regeneration; but, on the other hand, this does not occur during chronic injuries, in which myogenic regeneration is deregulated. Muscle degeneration, observed in chronic muscular injury diseases such as Duchenne muscular dystrophy (DMD), is in fact characterized by a gradual decline of regenerative potential.

DMD is the most severe muscular genetic disease in which the loss of dystrophin in the muscle leads to myofiber fragility and successive cycles of muscle necrosis and regeneration. Over time, the capacity of dystrophic muscle to regenerate becomes impaired, and muscle quality declines on account of increased fibrosis and adipose tissue deposition due to an abnormal persistence of FAPs. Indeed, the detrimental environment due to disease causes a decreased ability of MuSCs to replace degenerating muscles and an increase in the fibro-adipogenic activity of FAPs, the latter of which has proven to be a source of pro-atrophic and pro-fibrotic signals. In this dramatic context, the crosstalk between the different cell populations fails, and the regeneration ability of the entire tissue is hampered leading to muscle atrophy (Mozzetta et al., 2013; Madaro et al., 2018).

Several efforts have been devoted to the identification of effective treatments able to support the regeneration of skeletal muscle in both acute and chronic conditions. MSCs from different tissues are being studied for their ability to regenerate or repair skeletal muscle. In particular, MSCs are able to induce the proliferation and differentiation of resident MuSCs and are also able to act on the other cellular components of the muscle cell niche by reducing inflammation and infiltration.

In the following sections, we will describe evidence of the therapeutic effects of MSCs in different preclinical models of skeletal muscle injury. We will first focus on studies on acute muscle injury models in which skeletal muscle tissue is physiologically induced to activate a myogenic program and, then, on chronic pathologic damage associated with different diseases.

### Acute Muscle Injuries

Skeletal muscle is a tissue with a great regenerative capacity; but if profoundly scratched due to car accidents or sport injuries, it recovers only 50% in strength and 80% of its ability to shorten (Noonan and Garrett, 1999). Up to 20% of muscle mass loss can be compensated by the high adaptability and regenerative potential of skeletal muscle (Liu et al., 2018).

MSCs can be used to regenerate or repair skeletal muscle that has been damaged by acute injuries.

MSCs from the bone marrow (BM-MSCs) were the first and are the most studied. Intramuscular transplantation of BM-MSCs in rats with severe muscle injury has been shown to contribute to skeletal muscle healing by downregulating inflammatory cytokines levels [interleukin (IL)-1b, IL-6, TNF-α, and IFN-α] and by inducing anti-inflammatory cytokines (IL-10) (Helal et al., 2016). In addition, when compared with untreated controls, BM-MSC-treated muscles were characterized by the presence of regenerating myofibers and angiogenesis restoration (Helal et al., 2016). Moreover, BM-MSC transplantation has been shown to counteract the accumulation of fibrotic tissue in injured muscles by inhibiting the downstream signaling of the pro-fibrotic cytokine transforming growth factor beta 1 (TGF-β1) (Helal et al., 2016). Additionally, allogeneic BM-MSCs have also been shown to support the formation of new muscle fibers (Andrade et al., 2015) when directly injected in the muscles of rats with acute damage. The results showed an acceleration of muscle function recovery (Andrade et al., 2015).

Interestingly, in addition to BM-MSCs, adipose tissue-derived MSCs (AT-MSCs) and synovial membrane-derived MSCs (SM-MSCs) have also been shown to be active contributors to skeletal muscle regeneration (De La Garza-Rodea et al., 2012). More in detail, De la Garza-Rodea and collaborators demonstrated that all three types of MSCs were able to contribute to skeletal muscle regeneration in the cardiotoxin-injured mouse (CTX mouse), a model of acute damage in the skeletal muscle tissue (De La Garza-Rodea et al., 2012). However, they demonstrated enhanced effects of AT-MSCs on myofiber formation/regeneration when compared with BM-MSCs, and this was attributed to higher engraftment in the tibialis anterior muscles (De La Garza-Rodea et al., 2012).

The regenerative effects of both allogenic BM-MSCs and AT-MSCs were also described in the treatment of skeletal muscle laceration injury in Wistar rats (Moussa et al., 2020). In particular, both types of MSCs were shown to be effective and lasted up to 8 weeks post treatment, even if intramuscular injection of AT-MSCs displayed a stronger effect leading to an increase of myotube formation in parallel to a decrease of collagen deposition (Moussa et al., 2020). Similar results were obtained after immortalized BM-MSCs embedded within Pluronic F-127 hydrogel were locally injected in a muscle contusion murine model (Chiu et al., 2020). As a matter of fact, mice treated with BM-MSCs embedded in the hydrogel displayed numerous regenerating myofibers, as well as improved muscle strength, when compared with the control group (Chiu et al., 2020).

The long-term effects of the local injection of autologous BM-MSCs, combined with plastic surgery, to treat muscle necrosis were also recently investigated in a pig model of severe radiation burn (Linard et al., 2018). Very interestingly, BM-MSC treatment supported muscle regeneration even 1 year after surgery, leading to the restoration of myofiber diameter and density, stimulation of fast-twitch to slow-twitch fiber conversion, accelerated restoration of vascular structures, and regulation of M1/M2 macrophage balance in the muscle (Linard et al., 2018).

Intriguingly, transplantation of Wharton’s jelly-derived MSCs (WJ-MSCs) in a mouse model of skeletal muscle injury was shown to attenuate neutrophil-mediated acute inflammation post injury and to reduce fibrous tissue accumulation by modulating TGF-β1 levels in the muscle (Su et al., 2019). The effects of WJ-MSCs were also evaluated by Kwon and colleagues who reported anti-apoptotic properties of WJ-MSCs exerted in a mouse skeletal myoblast cell line (C2C12) (Kwon et al., 2016). They demonstrated that when co-cultured in serum-deprived with C2C12, WJ-MSCs secrete high levels of the chemokine CXCL1 (Chemokine C Motif Ligand 1) that was responsible for the anti-apoptotic effect of human WJ-MSCs (Kwon et al., 2016).

Taken together, these studies sustain the therapeutic efficacy of MSCs derived from different sources and demonstrate that they promote muscle regeneration in different animal models of acute muscle injury. Comparative studies have demonstrated the great effectiveness of MSCs in different directions: inducing new muscle fibers; decreasing inflammation by acting on cytokines, neutrophils, and macrophages; decreasing fibrosis by the modulation of TGF-β1 levels in muscles; increasing vascularization; and improving also muscle functionality. Very interestingly, a long-term beneficial effect upon MSC transplantation has also been observed, further supporting future translation into the clinical practice.

### Chronic Muscle Injuries

MSCs have been also applied as a therapeutic strategy to promote muscle regeneration in the presence of conditions due to genetic alterations (i.e., muscular dystrophies).

Seminal works have discovered that BM-MSCs have the intriguing potential to induce skeletal muscle regeneration in the mdx mouse, a model for DMD, in which mutations in exon 23 of the dystrophin gene lead to the protein deletion, thus mimicking the human pathology characterized by progressive muscle degeneration. As a matter of fact, when injected intraperitoneally into the mdx mouse, BM-MSCs, isolated from the femoral and tibial bone marrow of dystrophin/utrophin double-knockout mice, were able to improve disease symptoms by increasing locomotor activity and prolonging mouse survival (Maeda et al., 2017). Noteworthy, BM-MSC transplantation also increased the number of MuSCs and significantly decreased fibrosis in the diaphragm (one of the most affected organs in DMD). Furthermore, when MuSCs were co-cultured with BM-MSCs, an improvement of myotube differentiation was observed (Maeda et al., 2017).

The therapeutic potential of BM-MSCs in dystrophin/utrophin double-knockout mice was also previously reported (Li et al., 2011). BM-MSC administration via the caudal vein ameliorated the symptoms, strengthened the muscle functionality, and, noteworthy, induced the expression of dystrophin and utrophin genes in the treated mice (Li et al., 2011).

A recent work reported the beneficial effects of intravenous injection of WJ-MSCs in the mdx mouse model (Choi et al., 2020). Indeed, treatment determined a decrease of fibrosis and of the percentage of necrotic fibers (Choi et al., 2020). The authors also indicated the paracrine secretion of the matrix metalloproteinase-1 (MMP-1) as the key factor that exerted the anti-fibrotic effect of WJ-MSCs in the muscle (Choi et al., 2020).

The ability of AT-MSCs to promote skeletal muscle regeneration in dystrophin-deficient mice was also demonstrated (Pinheiro et al., 2012). Local injection of AT-MSCs improved muscle strength and resistance to fatigue, and this was mediated by modulation of inflammation and regulation of different genes involved in the regenerative process [i.e., myogenin, vascular endothelial growth factor (VEGF), TGF-β1, IL-6, IL-10, and IL-4] (Pinheiro et al., 2012).

Very interestingly, the effect of the CD146^+^ cell (i.e., pericytes) subpopulation of the stromal fraction of human adipose tissue was investigated and compared with that of AT-MSCs from the same sample. Weekly intraperitoneal transplantations of CD146^+^ cells in the DMD mouse model were able to sustain muscle regeneration for up to 8 weeks after transplantation, and this effect was stronger when compared with that of AT-MSCs (Gomes et al., 2018).

The potential of human dental pulp stem cells (DP-MSCs) and of human amniotic fluid stem cells (AF-MSCs) was also assessed in an immune-compromised mdx/SCID model (Pisciotta et al., 2015). MSCs were pre-committed *in vitro* toward the myogenic lineage by means of a DNA demethylation treatment, in both the presence and absence of CM from differentiated C2C12 cell cultures, that induced the expression of specific myogenic commitment markers (i.e., myogenin, myosin heavy chain, and desmin). Pre-differentiated DP-MSCs and AF-MSCs were then injected intramuscularly in mice, and both cell types were able to restore dystrophin expression and slow down muscle degeneration by exerting pro-angiogenic and anti-fibrotic effects (Pisciotta et al., 2015).

MSCs have also been tested in sarcoglycanopathies, another chronic muscular disorder (Shabbir et al., 2009). These diseases result from the absence of sarcoglycan proteins whose role is to connect the cytoskeleton of muscle fibers to the sarcolemma in order to maintain structural integrity of the myofibers (Gao et al., 2015). Indeed, Shabbir and colleagues demonstrated that intramuscular transplantation of human or pig BM-MSCs in delta-sarcoglycan-deficient dystrophic hamsters was able to significantly decrease CD45 and NFkB expression. In addition, MSC treatment was able to decrease malondialdehyde (MDA) expression, the final product of lipid peroxidation induced by inflammation that characterizes dystrophic muscles, similar to that of wild-type mice (Shabbir et al., 2009).

Based on these findings, the authors concluded that the immunomodulatory properties of MSCs can be exploited as a possible therapy for chronic inflammatory conditions such as muscular dystrophies (Shabbir et al., 2009).

Altogether, several studies have shown how MSCs derived from different sources, injected both locally and systemically, can also exert beneficial effects in severe forms of muscular dystrophies by acting at different levels to hamper muscle degeneration.

### Atrophic Muscle

Muscular atrophy can be a physiological process due to aging or long-term immobilization, or it can be a consequence of pathogenic conditions such as spinal muscular atrophy (SMA) or other chronic diseases like muscular dystrophies, amyotrophic lateral sclerosis (ALS), cancer, acquired immunodeficiency syndrome (AIDS), and diabetes (Bonaldo and Sandri, 2013). Muscular atrophy is characterized by the loss of muscle mass due to myofiber death, change in fiber types and myosin isoforms, reduction of cell cytoplasm, loss of cellular organelles, and, in particular, protein degradation that exceeds the synthesis of new proteins.

Kim and colleagues demonstrated how human MSCs isolated from different sources (bone marrow, adipose tissue, and umbilical cord) can induce muscle regeneration after transplantation into the soleus of rats with hindlimb suspension-induced muscle atrophy (Kim et al., 2015). In particular, the authors observed activation of the phosphoinositide-3-kinase (PI3K)/Akt pathway and consequently a reduction of muscle-specific RING finger protein-1 (MuRF-1) and atrophy F-box (MAFbx/Atrogin-1) expression in rats treated with MSCs (Kim et al., 2015).

ALS is a serious progressive neurodegenerative pathology characterized by the loss of motor neurons with consequent atrophy and loss of muscle movements (Hardiman et al., 2017). Numerous studies conducted in mice with the Gly93Ala mutation in the SOD1 gene (SOD1-G93A mice), which leads to the development of ALS symptoms (progressive loss of muscle strength, onset of the disease at about 5 months, and a life span of about 6 months) (Julien and Kriz, 2006), have demonstrated that BM-MSCs induce important therapeutic effects (Zhao et al., 2007; Vercelli et al., 2008; Zhou et al., 2013). Different works highlighted how BM-MSC transplantation in ALS mice improves disease phenotype, delays disease progression, and induces partial recovery of the motor function (Zhao et al., 2007; Vercelli et al., 2008; Gugliandolo et al., 2019).

In particular, it has been shown that intravenous injection of human BM-MSCs injected into SOD1-G93A mice was able to delay the development of pathology-related symptoms and to promote survival when compared with untreated and vehicle-injected mice (Zhao et al., 2007). Moreover, the functions of neuromotors were also analyzed, and again, significant differences were found. In particular, when compared with untreated mice, those that received human BM-MSCs lost motor function almost a month after treatment and had a higher number of motor neurons (Zhao et al., 2007). Furthermore, human BM-MSCs prevented the loss of peripheral motor nerves, and reinnervation took longer for treated mice.

Zhang and collaborators also demonstrated that multiple intrathecal injections of human BM-MSCs induced therapeutic effects in SOD1-G93A mice by enhancing motor performance, decreasing motor neuron loss, and increasing survival through the inhibition of the inflammatory response as shown by downregulation of TNF-α and iNOS protein levels (Zhang et al., 2009; Zhou et al., 2013).

Another study also demonstrated that when transplanted into the lumbar spinal cord of SOD1-G93A mice, BM-MSCs were able to migrate in the lumbar spinal cord, prevent microglial activation, and delay disease onset associated with a decrease in the number of motor neurons, thus globally leading to a better muscle performance (Vercelli et al., 2008).

The striking effectiveness of AT-MSCs for ALS has also been demonstrated. Systemic injection of AT-MSCs into the SOD-1 mutant mouse was able to modulate the secretome of local glial cells and increase glial-derived neurotrophic factor (GDNF) levels, which consequently led to neuroprotection and increased the number, survival, and functionality of motor neurons (Marconi et al., 2013).

Taken together, administration of MSCs, and especially of BM-MSCs, in ALS models is effective in decelerating disease symptoms as well as in sustaining muscle innervation, acting on both muscle and motor neuron sides.

## The Therapeutic Potential of the Mesenchymal Stromal Cell Secretome

In recent years, the regenerative potential of MSCs has not been attributed so much to the ability of these cells to engraft into target tissue and differentiate but to their ability to secrete factors capable of supporting tissue regeneration through activation of resident cells.

CM-MSCs have been shown to be a valid alternative to its cellular counterpart (MSCs) by numerous *in vitro* and *in vivo* studies, which describe similar beneficial effects between the two (Gnecchi et al., 2006; Aslam et al., 2009; Rossi et al., 2012; Goolaerts et al., 2014).

Different *in vitro* studies have shown that CM-MSC affects different immune cell populations of the innate (macrophages, dendritic cells, neutrophils, and natural killer cells) and adaptive (T and B cell) immunity. For example, CM-MSCs obtained from different tissues, such as bone marrow, adipose tissue, and muscle tissue, inhibit T-cell proliferation (Di Nicola et al., 2002; Keyser et al., 2007; Hegyi et al., 2012; Lang et al., 2018), support the expansion of T regulatory cells (Nasef et al., 2007; Yang et al., 2009; Sattler et al., 2011; Tasso et al., 2012), and inhibit B-cell proliferation (Augello et al., 2005; Corcione et al., 2006) and B-cell differentiation (Asari et al., 2009). Furthermore, CM-MSCs have been shown to induce a phenotype and functional switch of monocytes toward macrophages with anti-inflammatory, M2-like features (Onishi et al., 2015; Pischiutta et al., 2016; Magatti et al., 2017; Giampà et al., 2019), to inhibit natural killer cell proliferation and cytotoxicity (Rasmusson et al., 2003; Sotiropoulou et al., 2006), and to inhibit dendritic cell differentiation (Nauta et al., 2006; Djouad et al., 2007; Ramasamy et al., 2007; Li et al., 2008). Moreover, the administration of CM-MSC has been shown to induce therapeutic effects in a wide variety of disease models, such as sepsis (Németh et al., 2009), and to support the repair of several tissues such as the liver (Zagoura et al., 2012), lungs (Ray et al., 2003; Cargnoni et al., 2012; Turner et al., 2013), skin (Lee et al., 2011), and heart (Mirabella et al., 2011) and moreover, it possesses neuroprotective and neurotrophic abilities (Mead et al., 2014; Caseiro et al., 2016; Giampà et al., 2019).

The advantages of using CM-MSCs lie in its composition of soluble factors and EVs. In particular, EVs are very attractive for a cell-free therapeutic approach in regenerative medicine since they bypass some undesirable side effects of MSCs, such as their ability to enhance tumor growth by chemokine or cytokine secretion (Liu et al., 2011; Tsai et al., 2011; Yan et al., 2012), and their inability to proliferate overcomes the tumorigenesis potential of MSCs (Rani et al., 2015; Mardpour et al., 2019).

The use of MSC-EVs as a therapeutic approach is advantageous also for other reasons: (i) the ability to migrate specifically into the target organ while MSCs often become entrapped in the microvasculature leading to a high risk of thrombosis (Gowen et al., 2020; Zhao et al., 2020); (ii) the systemic injection of EVs has an improved safety profile and lower immunogenicity with any histopathological changes or increases of liver transaminases or cytokine levels (Koniusz et al., 2016; Zhu et al., 2017; Leavitt et al., 2019; Saleh et al., 2019; Shiue et al., 2019); (iii) EVs are a putative delivery system of genes, drugs, enzymes, and RNAs, and they can be engineered to target specific cells or pathways (Yeo et al., 2013; Pascucci et al., 2014; Tian et al., 2018; Asgarpour et al., 2020; Conceição et al., 2021). All these characteristics make MSC-EVs promising tools for cell-free therapeutics.

### Therapeutic Effects of the Mesenchymal Stromal Cells Secretome in Muscle Regeneration

As for other pathologies, it has been proven that when locally or systemically injected, MSCs activate muscle resident cells due to the release of paracrine factors, such as VEGFa, improving muscle regeneration and/or modulating muscle inflammation and muscle fibrosis (Pinheiro et al., 2012; Secco et al., 2013; Valadares et al., 2014).

In the context of skeletal muscle diseases, Assoni and colleagues demonstrated *in vitro* that CM from adipose tissue, skeletal muscle, and uterine tube MSCs has the ability to modulate apoptosis of dystrophic myoblasts, also enhancing cell migration and proliferation (Assoni et al., 2017). Through proteomic profiling of the CM, they also demonstrated the great variability of proteins from the different sources analyzed but also highlighted common enriched pathways related to extracellular matrix organization, axon guidance, antigen processing, metabolic processes, and positive regulation of nitric oxide (Assoni et al., 2017).

Another study demonstrated that the transplantation of CM-WJ-MSCs showed better results on inflammation and collagen deposition in muscle tissue than transplantation of the source cells (Pereira et al., 2014). These effects were due to the presence of cytokines and growth factors involved in the suppression of local immune system, such as hepatocyte growth factor (HGF) and IL-10, in the improvement of angiogenesis, such as fibroblast growth factor (FGF) and VEGFA, and in the inhibition of scar formation (Chen et al., 2008; Li et al., 2009; Jackson et al., 2012).

In a different study, Kim and collaborators showed that injection of human CM-UC-MSCs in a mouse model of muscle atrophy induced recovery of the muscle mass through the activation of PI3K/Akt pathway (Kim et al., 2016). In addition, using an antibody-based protein array, the authors characterized factors released by human umbilical cord-derived MSCs (UC-MSCs) (Kim et al., 2016). Interestingly, several regulators of muscle regeneration were identified: ectodysplasin-A2, thrombospondin-1, IL-6, monocyte chemoattractant protein-1 (MCP-1), dickkopf-related protein 1 (DKK1), HGF, VEGF, FGF7, tissue inhibitor of metalloproteinase 1 (TIMP-1), SMAD family member 4 (SMAD4), macrophage inflammatory protein 2 (MIP-2), activin A, insulin-like growth factor-binding protein (IGFBP)-related protein 1, and MMP-1 (Kim et al., 2016).

These seminal works clearly demonstrate that the efficacy of MSCs in supporting skeletal muscle regeneration is attributed to secreted factors that exert a bioactive effect in damaged muscles.

### Therapeutic Effects of Extracellular Vesicles Derived From Mesenchymal Stromal Cells in Muscle Regeneration

MSC-EV-derived therapeutic approaches have been exploited in several pathologies, such as liver diseases (Lou et al., 2015; Qu et al., 2017; Chen et al., 2018; Shao et al., 2020; Zhang et al., 2020), brain diseases including brain tumors (Xin et al., 2012, 2013; Xu et al., 2019; Allahverdi et al., 2020), cardiac dysfunctions, and myocardial infarction (Feng et al., 2014; Yu et al., 2015).

The therapeutic potential of MSC-EVs in supporting skeletal muscle regeneration in the context of muscle diseases has also been evaluated. Nakamura et al. (2015) described the *in vitro* beneficial effect of EVs isolated from BM-MSCs on myogenesis and angiogenesis of C2C12 myoblasts and HUVECs, respectively. The authors confirmed these data in the CTX mouse model, in which they performed an intramuscular injection of MSC-EVs and observed an increase of muscle cross-sectional area, a decrease of fibrotic area, and an improvement of capillary density (Nakamura et al., 2015). The interest toward MSC-EVs as an efficacious treatment for degenerative neuromuscular diseases has also escalated due to the fact that EVs are lipid vesicles of endocytic origin that can cross the blood–brain barrier (BBB) in a non-invasive manner (Zhuang et al., 2011; Zhou et al., 2014). Several studies have reported that the beneficial effects are associated with EV cargo. In particular, a comparison of miR content in MSC-EVs and in CM-MSCs has revealed several miRs in common and others exclusive for MSC-EVs (Nakamura et al., 2015). For example, miR-21, an anti-apoptotic miR, was present in both the MSC-EVs and MSC-CM, but as already reported by others, miRs encapsulated in EVs appeared to have enhanced functions when compared with miRs released in the total CMs (Shimbo et al., 2014; Nakamura et al., 2015). The analysis of miRs cargo showed the presence of myogenic miRs, such as miR-1, miR-133, and miR-206, but also of miR-494, which has been shown to induce a protective effect against ischemia-induced cardiac injury (Wang et al., 2010; Nakamura et al., 2015). Lo Sicco and colleagues investigated the angiogenic effect of EVs isolated from AT-MSCs in a mouse model of muscle damage (Lo Sicco et al., 2017). They implanted matrigel plugs containing MSC-EVs in mice and observed that 3 weeks after the implantation, there was an increase of vessels along the periphery of the plugs (Lo Sicco et al., 2017). The described effect of MSC-EVs was associated with the high expression, in their cargo, of the angiogenic factors platelet and endothelial cell adhesion molecule (PECAM) and VEGFA (Lo Sicco et al., 2017). Moreover, EVs isolated from MSCs cultured in hypoxic conditions were able to upregulate the expression of several miRs implicated in muscle repair, in particular miR-223, miR-146b, miR-126, and miR-199a (Lo Sicco et al., 2017). By *in vitro* and *in vivo* experiments, MSC-EVs, especially when isolated from MSCs cultured in hypoxic conditions, were able to modulate inflammation by regulating macrophage polarization and accelerating muscle regeneration (Lo Sicco et al., 2017). Concordantly, others described the beneficial effects of the secretome (the whole CM and EV fraction) of human AF-MSCs on muscle homeostasis, highlighting their anti-inflammatory activity, their ability to enhance proliferation, and their capacity to protect against cellular senescence in the CTX mouse model (Mellows et al., 2017). In particular, the beneficial effect observed was mediated, at least in part, through the repression of NF-κB pathway, which appears stronger in MSC-EVs compared with the whole CM. Interestingly, the pro-regenerative effect of MSC-EVs in CTX mice was also associated with their miR content, which was predicted to promote angiogenesis, proliferation, migration, differentiation, autophagy, apoptosis, and inflammation (Mellows et al., 2017). A comparison of the effects of the whole CM and EVs from AF-MSCs in the CTX mouse model revealed very few differences in muscle regeneration. CM-AF-MSCs increased regenerating fiber size, the number of capillaries/fibers, and the level of committed muscle stem cells, while AF-EVs increased the regenerating fiber size and the number of capillaries/fiber (Mellows et al., 2017).

On the other hand, the recent comparison of the whole secretome with the EV fraction with AT-MSCs showed differences in protein and miR expression leading to a different impact from secretome and/or EVs on biological processes. Several factors (both proteins and miRs) are described as potentially involved in different processes (Mitchell et al., 2019). For example, the whole secretome but not the EV fraction influenced senescence, while EVs but not the whole secretome impacted inflammation (Mitchell et al., 2019). Notably, Mitchell and colleagues highlighted the greater effect of EVs in muscle repair in acute damage conditions in CTX mice. Indeed, by studying the miR contents in MSC-EVs, they observed the presence of anti-inflammatory (i.e., miR-let7 family), pro-regenerative (i.e., miR-145), and angiogenic miRs (i.e., miR-23a), all able to improve muscle regeneration in CTX mice (Mitchell et al., 2019).

Local injection of exosomes isolated from the CM of BM-MSCs was very recently shown to accelerate the recovery of contractile function of muscles in a rat model of muscle injury (Iyer et al., 2020). Treatment stimulated the formation of new fibers and modulated the expression of genes involved in inflammation, fibrosis, and myogenesis mechanisms (Iyer et al., 2020). Furthermore, another study demonstrated that intramuscular injection of exosomes from BM-MSCs after muscle contusion in mice modified the polarization status of macrophages, alleviated the inflammatory reaction, reduced fibrosis size, promoted muscle regeneration, and improved fast-twitch and tetanus strength (Luo et al., 2020).

The therapeutic effects of placenta-derived MSCs (PL-MSCs) and their secreted EV exosomes were assessed *in vitro* in myoblasts isolated from DMD patients and mdx mice and *in vivo* by intramuscular transplantation of PL-MSCs in mdx mice (Bier et al., 2018). In particular, both PL-MSCs and their EVs promoted fusion and differentiation of human muscle cells from DMD patients, as well as decreased the expression of TGF-β and thus the fibrogenic differentiation of DMD myoblasts. MiR-29 was identified as a mediator of this effect since it has been associated with various pathological pathways in DMD, and it is downregulated in myoblasts from DMD patients compared with muscle cells from healthy donors (Bier et al., 2018). Intriguingly, both PL-MSCs and their EVs induced the dystrophin homolog utrophin in C2C12 cells and in human myoblasts derived from healthy donors and DMD patients. Intramuscular transplantation of PL-MSCs and EVs in mdx mice showed comparable effects; in fact, both treatments inhibited fibrosis and inflammation (Bier et al., 2018). Also, local injections of EVs isolated from UC-MSCs or systemic administration of murine BM-MSC-EVs in mdx mice counteracted DMD pathology. Both treatments induced the recovery of muscle function, the decline of creatinine kinase (CK) blood levels, and the decrease of muscle fibrosis and inflammation due to the re-localization of the dystrophin-associated protein complex (DAPC) (Leng et al., 2020).

Our recent studies have also demonstrated how miR content of EVs derived from muscular interstitial MSCs, FAPs, could play a pivotal role in muscle regeneration (Sandonà et al., 2020). We demonstrated that EVs mediate the communication between FAPs and MuSCs in dystrophic mice; and additionally, we reported the first evidence of pharmacological treatment ability to fine-tune EV cargo, enhancing their regenerating effects on muscle fibers (Sandonà et al., 2020). The pharmacological treatment of mdx with an epigenetic drug, histone deacetylase (HDAC) inhibitor [HDACi; i.e., Trichostatin A (TSA)], induced a significant upregulation of 14 miRs inside the EVs released by FAPs of dystrophic mice (miR-206, miR-542, miR-449a, miR-342, miR-320, miR-192, miR-423, miR-376a, miR-145a, miR-224, miR-30a, miR-494, miR-29a, and miR-7b), which are mainly involved in muscle regeneration and muscle homeostasis (Sandonà et al., 2020). Among them, miR-206 was found to be the most upregulated following HDACi treatment and was found to be crucial to confer the ability to drive muscle regeneration, and to reduce fibrotic tissue deposition and muscle inflammation, to EVs injected in dystrophic muscles. This was confirmed by antagomiR inhibition of miR-206 expression in EVs, which led to the loss of the ability of EVs to impact muscle regeneration and muscle fibrosis but did not affect muscle inflammation (Sandonà et al., 2020). In addition, the inhibition of the expression of miR-145a, another HDACi-induced miR in EVs, reduced inflammation in the muscles of mdx mice. Therefore, we concluded that the specific assembly of miRs inside the EVs is fundamental to reveal their therapeutic effect in DMD (Sandonà et al., 2020).

Interestingly, the therapeutic efficacy of MSCs secreted vesicles has also been recently demonstrated in muscle atrophy. In particular, EVs from human UC-MSC (hUC-MSC-EVs) injected in rats with sciatic nerve defects were capable of restoring hind leg muscle mass thanks to extensive muscle innervation (Ma et al., 2019). The hUC-MSC-EVs promoted the nerve regeneration by the modulation of the inflammation, as observed by the downregulation of IL−6 and IL−1β and the up−regulation of IL−10, improving functional recovery. There is also reported evidence that the increment of muscle mass in atrophic muscles could be restored by the injection of MSC-EVs in injured muscles of rat with massive rotator cuff tear (MRCT) (Wang et al., 2019). In this model, EVs inhibited macrophage migration and the release of pro-inflammatory cytokines, preventing secondary muscle damage; moreover, vesicles were able to reduce the apoptosis of tenocytes and myocytes and to increase myogenesis of endogenous stem cell residing in the muscle (Wang et al., 2019).

Therapeutic approaches with MSC-EVs have also been tested on other chronic pathologies such as ALS. There are new and promising therapeutic approaches for ALS using MSC-EVs, in particular those isolated from AT-MSCs. AT-MSCs possess neuroprotective properties mediated by their EVs. EVs derived from AT-MSCs and in particular exosomes could be used for therapeutic approaches for ALS because they are capable of acting on different hallmarks of the disease. Indeed, an *in vitro* study using the NSC-34 motorneuron cell line overexpressing hSOD1 has shown that AT-MSC-EVs prevented H_2_O_2_-induced damage and increased cell viability (Bonafede et al., 2016).

The subsequent proteomic analyses of AT-MSC-derived exosomes revealed that it contains proteins that affect pathways crucial for ALS pathology, such as cell adhesion, apoptosis, response to oxidative stress, and PI3K/Akt signaling pathway (Bonafede et al., 2019). The neuroprotective effect of AT-MSC-derived exosomes in ALS could be associated with the presence of the ribonuclease RNAse4, a protein mutated in ALS patient, which is known to display angiogenic, neurogenic, and neuroprotective activities (Li et al., 2013; Bonafede et al., 2019; Padhi and Gomes, 2019). With the use of an *in vitro* model of ALS, the transfer of SOD1 and SOD3 through exosomes destroyed free superoxide radicals generating a protective effect replacing the enzymatic function of mutated SOD1, thus improving response to oxidative stress. In addition, Igf1, which activates the PI3K/Akt signaling pathway and binds Igf1R promoting proliferation and inhibiting apoptosis, was also found inside AT-MSC-derived exosomes (Bonafede et al., 2019). Other *in vitro* studies on neural stem cells (NSCs) isolated from the SOD1-G93A ALS mouse model have demonstrated that treatment with AT-MSC-EVs could reduce the increase of SOD1 aggregation in the cytoplasm of cells (Lee et al., 2016). Moreover, EV treatment had also effects on mitochondrial defects: NSCs treated with AT-MSC-EVs showed an increase in mitochondrial protein expression, such as p-CREB/CREB and PGC1α involved in the activation of mitochondrial biogenesis and have been reported to be abnormally expressed in ALS (Kong and Xu, 1998; Lee et al., 2016).

The recent available literature clearly suggests that the intravesicular portion of CM-MSCs has therapeutic implications for musculoskeletal diseases and, at the same time, assesses the feasibility of using EVs in treatment of diseases that have no cure to date.

The deep investigation of the identity of EV cargo in terms of proteins, mRNA, and miRNAs can be harnessed therapeutically in the more immediate future by engineering vesicles for specifically delivering therapeutic components to the target tissue.

## Clinical Trials in Humans

Thanks to their regenerative potential, MSCs have been tested in different clinical trials for cardiovascular, neurological, and immunological diseases, among others (Chen et al., 2004; Bang et al., 2005; Lazarus et al., 2005; Ringden et al., 2006; Ripa et al., 2007; Markert et al., 2009; Lee et al., 2012; Rajput et al., 2015; Pezzi et al., 2017; Kim et al., 2018; Winkler et al., 2018). Despite several advantages of using MSCs in clinical trials, most of them have failed to reach primary endpoints. The therapeutic ineffectiveness resides mostly on the age of the MSC donor, on the different MSC isolation and culture methods, on the various MSC administration routes, and on the MSC recipient (host) (Yukawa et al., 2012; Siegel et al., 2013; Stubbendorff et al., 2013; Pezzi et al., 2017).

For example, cell isolation methods can yield non-homogenous cell populations that can consequently affect clinical outcome. In addition, long-term culture has been shown to decrease differentiation ability (Drela et al., 2019), increase malignant transformation of MSCs from BM (Røsland et al., 2009; Drela et al., 2019), and reduce engraftment *in vivo* (Bonab et al., 2006; Tolar et al., 2007; Yang et al., 2018).

Although several studies have shown the efficacy of both MSCs and their secretome in preclinical models of muscle diseases, there are very few clinical trials.

With the use of the key terms “muscle” and “mesenchymal cells” and selecting only studies concerning the use of MSCs in muscular diseases, eight clinical trials are registered on ClinicalTrial.gov^1^. Amongst these, six are in DMD patients, one in patients with facioscapulohumeral dystrophy (FSHD), and one in Werdnig–Hoffman patients. Four trials involved the use of umbilical cord MSCs, two used adipose-derived MSCs, and the remaining two used BM-MSCs. There are no results available for these eight selected studies on ClinicalTrial.gov^1^.

Twenty-two approved and registered clinical trials were found on www.clinicaltrials.gov using the search terms “ALS” and “mesenchymal cells”; and they also evaluated the efficacy of MSCs, isolated from different sources, in ALS patients. The results were recently reviewed in detail by Gugliandolo and colleagues (Gugliandolo et al., 2019).

Very interestingly, a phase 2 clinical trial (ClinicalTrials.gov Identifier: NCT03406780) investigated the safety and efficacy of a cell therapy called CAP-1002 in DMD patients. CAP-1002 is a therapeutic product composed of allogenic cardiac progenitor cells, namely, cardiosphere-derived cells, and it has been previously demonstrated that intracoronary infusion of CAP-1002 is feasible, safe, and potentially effective in DMD patients (Taylor et al., 2019). These cardiospheres have been proposed to act through the secretion of different growth factors and exosomes exerting anti-inflammatory, anti-fibrotic, and regenerative actions in the target organ (Taylor et al., 2019).

In the last couple of years, different publications have reported clinical trials results. For example, the suitability, safety, and efficacy of intra-arterial and intramuscular administration of allogeneic WJ-MSCs in ambulatory and non-ambulatory DMD patients were found be well tolerated, ameliorated symptoms, and slowed the progression of the disease in the first year following the administration (Dai et al., 2018). Furthermore, the feasibility of treatment with BM-MSCs in patients with DMD (Sharma et al., 2014; Klimczak et al., 2020) and with ALS (Nabavi et al., 2019) has been also evaluated. In particular, intravenous and intrathecal injections of autologous BM-MSCs in ALS patients were found to be safe and feasible (Nabavi et al., 2019). Klimczak and colleagues tested the effects of the intramuscular co-transplantation of BM-MSCs and myogenic progenitor cells in three DMD patients, based on the rationale that both cell types can merge with damaged myofibers to regenerate skeletal muscle and from the knowledge that BM-MSCs are involved in myogenesis due to their ability to differentiate into myoblasts and to inhibit chronic inflammation that characterizes DMD (Klimczak and Kozlowska, 2016; Klimczak et al., 2018). They demonstrated that BM-MSCs had immunomodulatory properties that supported the regenerative potential of the myogenic precursors *in vivo* (Klimczak et al., 2020).

Finally, intravenous administration of UC-MSCs in patients affected by Becker muscular dystrophy was found to be safe and determined an increase of muscle strength, a result that is interestingly more evident in patients with a shorter course of disease (Li et al., 2015).

## Conclusion

The incredible and multifaceted properties of MSCs have attracted great scientific interest for the possible development of numerous therapeutic applications, making them the most commonly used cells, especially in the regenerative medicine field. They can be obtained from many different adult tissues and, for the most part, are easy to isolate and culture. When tissues are damaged, administrated MSCs have the ability to migrate to the site of injury and to release molecules (growth factors, cytokines, and EVs) that establish a favorable microenvironment and promote tissue homeostasis that supports or directly favors regeneration by the induction of pleiotropic effects: anti-inflammatory, immune-modulating, anti-fibrotic, angiogenic, and anti-apoptotic (Chapel et al., 2003; Caplan, 2009; Singer and Caplan, 2011; Le Blanc and Mougiakakos, 2012; Bronckaers et al., 2014).

In this review, we have emphasized the current knowledge about MSCs and how MSCs derived from different origins are widely used in the treatment of various diseases with a particular focus on muscular diseases. In the last decade, we have learned that MSCs act through paracrine factors, and in particular through the EVs they release. For all these reasons, we have provided insights into the possible therapeutic employment of the MSCs with a special attention to their secretome and EVs as novel off-the-shelf approaches for muscle regeneration.

Although different studies described the ability of MSCs to target specific damaged tissues, long-term engraftment is not often observed (Toma et al., 2002), and several alternative mechanisms have changed the “cell replacement theory” behind the beneficial effect of MSCs of promoting tissue repair.

**FIGURE 1:**
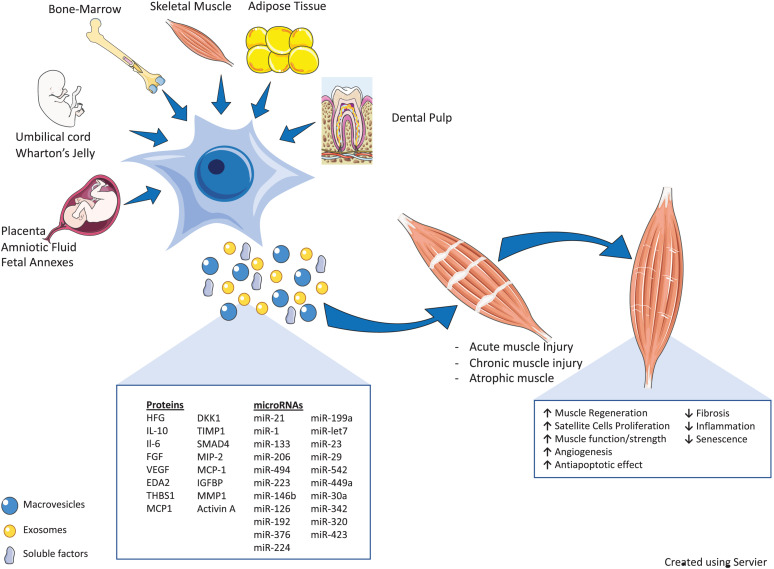
Therapeutic properties of mesenchymal stromal cells (MSCs) and their secretome [soluble factors and extracellular vesicles (EVs)] in various muscle injury models. MSCs derived from different tissues participate in skeletal muscle tissue regeneration, directly or through their secretome (soluble factors and EVs). The cartoon reports the bioactive fraction of the MSC secretome (cytokines, miRs, and growth factors) and the main regenerative processes induced by these factors, as discussed in the main text (↑, upregulation/induction; ↓, downregulation/inhibition). The schematic art pieces used in this figure were provided by Servier Medical art (https://smart.servier.com). Servier Medical Art by Servier is licensed under a Creative Commons Attribution 3.0 Unported License.

Numerous recent preclinical and clinical studies here discussed demonstrated that the therapeutic effect of MSCs secretome in skeletal muscle regeneration could be partly due to secreted EVs, which can mirror the therapeutic effect of their parent cells. We have discussed in detail how EVs released by MSCs are able to mediate intercellular communication that is translated into pleiotropic actions generating therapeutic potential through the transfer of active molecules, also in different muscle injury models (Figure 1).

The use of MSC-CM and in particular of MSC-derived EVs as a cell-free therapy is quickly developing as a promising option that could bypass the safety concerns associated with the use of live cells, ethical concerns associated with the origin of cells, and also immune-compatibility issues.

The MSC secretome is considered a potential bioactive pharmaceutical component, in which its vesicular portion, containing genetic information transmitted between cells of different types, is promising as a drug delivery system mainly due to homing capabilities, thereby opening an opportune window to specific and targeted compound (drugs, proteins, etc.) release into damaged lesions (Bari et al., 2018). A secretome-based approach should also minimize biological variability, allow precise dosing, and thus lead to the development of safe and effective therapeutic strategies with possibly predictable outcomes.

EVs isolated from MSCs constitute the best alternative to cell-free therapy due to low immunogenicity, high biocompatibility, and low cytotoxicity to tissues. They can also be used for nano-regenerative medicine since they can be engineered to target specific cells or tissues and can work as drug carriers.

We and others demonstrated the beneficial effects and efficacious employment of EVs in reducing muscle injury effects and enhancing tissue repair (Ma et al., 2019; Wang et al., 2019; Sandonà et al., 2020; Figure 1). The alteration in the content of these vesicles leads to a miscommunication between cells in the diseased muscles (i.e., dystrophic muscles) and alters their behavior. This study showed the ability of MSC-EVs to transfer the benefits of drugs without causing the unwanted systemic side effects of these treatments, bringing new hope to regenerative medicine for DMD (Sandonà et al., 2020).

Although there is a large body of evidence that has demonstrated the regenerative capacity of MSCs and their secretome in disease models of acute injury, chronic damage, and atrophy of the skeletal muscle, to date, little clinical evidence is reported in patients and is anyhow limited to the use of MSCs.

Unfortunately, clinical applications of EVs remain challenging due to lack of standardized protocols to produce vesicles for therapeutic use. There are open debates in the EV community (ISEV) about the diversity and preparation of MSCs and consequently about the methods of EV isolation and purification. These points, together with the lack of standardized quality assurance assays, and limited accuracy of *in vitro* and *in vivo* functional assays can affect the reproducibility of research results. In addition to these obstacles, low yield (Shao et al., 2018) and heterogeneity (Pegtel and Gould, 2019) need to be urgently addressed (Ayers et al., 2019).

A full understanding of the potential and efficacy of MSCs, and especially of their products, to support muscle regeneration will be a breakthrough for regenerative medicine that will identify new molecules for the repair and regeneration of skeletal muscle tissue.

## Author Contributions

MS, LDP, FE, AV, and VS: writing—original draft preparation. ARS, MS, LDP, OP, and VS: writing—review and editing. OP and VS: supervision. All authors final approval of the manuscript.

## Conflict of Interest

The authors declare that the research was conducted in the absence of any commercial or financial relationships that could be construed as a potential conflict of interest.

## Footnotes

1 https://www.clinicaltrials.gov/
